# Abnormal left ventricular geometry is prevalent in asymptomatic patients with established rheumatoid arthritis compared with those with early disease and healthy controls

**DOI:** 10.1186/1532-429X-17-S1-P297

**Published:** 2015-02-03

**Authors:** Bara Erhayiem, Lesley-Anne Bissell, Adam K McDiarmid, Peter P Swoboda, Ananth Kidambi, David P Ripley, Tarique A Musa, Laura E Dobson, Pankaj Garg, Paul Emery, Jacqueline Andrews, John P Greenwood, Maya H Buch, Sven Plein

**Affiliations:** 1 Leeds Institute of Rheumatic & Musculoskeletal Medicine, University of Leeds, Leeds, UK; 2 Multidisciplinary Cardiovascular Research Centre & Leeds Institute for Cardiovascular and Metabolic Medicine, University of Leeds, Leeds, UK

## Background

Rheumatoid arthritis (RA) is associated with increased incidence of cardiovascular disease (CVD) and heart failure (HF) compared to the general population. CMR studies in the general population demonstrate that increases in left ventricular (LV) mass and early LV structural changes are predictors of HF in asymptomatic patients. Previous studies have reported conflicting data on LV geometry in heterogeneously sampled RA populations.

## Objectives

To investigate the influence of disease duration on LV geometry using CMR by assessing and comparing two distinct RA populations; a well-defined population of newly diagnosed, treatment-naive RA and an established disease group.

## Methods

Seventy-five patients underwent CMR at 3.0T (Philips Achieva TX). None had known CVD or diabetes. 30 were RA patients fulfilling 2010 ACR/EULAR classification criteria, with symptoms for less than 1 year, no therapy with disease modifying anti-rheumatic drugs, disease activity score (DAS28) ≥3.2 and at least one poor RA prognostic factor. They were age-matched with 30 RA patients with disease duration >5yrs. 15 healthy controls were age-matched. Standard balanced steady state free precession cine images were acquired and LV dimensions calculated offline. Tissue-tagging for strain analysis was acquired using a spatial modulation of magnetization pulse sequence.

## Results

Participant characteristics can be seen in Table 1. Age, sex, blood pressure and body size were not different between the 3 groups. The established RA group had excellent disease control with median(IQR) CRP 0.0mg/L(5.3) and DAS28 2.0(1.7). CMR measurements are in table 2. LV systolic performance, using LVEF and Ecc, was normal and similar in all groups. LV geometry was similar between early RA patients and healthy controls (p-values for LVEDV, LV Mass and LV Mass/LVEDV being 0.19, 0.51 and 0.11 respectively). Indexed LV mass and LV mass/LVEDV were lower in the established RA group compared to the early RA group (Mean±SD, 33g±8/m^2^ versus 43±9g/m^2^, p<0.01 and 0.44±0.09g/ml versus 0.54±0.11g/ml, p<0.01 respectively).

**Table 1:** Participant characteristics according to RA status.

Characteristic	Early RA n = 30	Established RA n = 30	Healthy Control n = 15	p-value
Age, years	51±8	52±7	51 ± 8	0.81*

Female gender; n (%)	34 (65)	15 (50)	7 (47)	0.14*

Body mass index, kg/m^2^	27±5	26±4	25±3	0.48*

Body surface area, m^2^	1.9±0.2	1.8±0.2	1.9±0.2	0.35*

Pulse pressure, mmHg	51±11	57±14	55±14	0.34*

CRP, mg/L, median (IQR)	6.4 (34)	0.0 (5.3)	-	**<0.01**

ACPA, n (%)	26 (87)	24 (80)	-	0.19

RF, n (%)	25 (83)	21 (70)	-	0.14

DAS28, median (IQR)	5.5 (1.7)	2.0 (1.7)	-	**<0.01**

Data presented as mean and standard deviation unless otherwise stated. *Appropriate parametric/non-parametric tests applied for 3-way comparison after normality assessments. ACPA; anti-citrullinated peptide antibody, CRP; C-reactive protein, DAS; Disease activity score, RF; Rheumatoid factor.

**Table 2:** CMR findings between RA patients and controls.

Measurement	Early RA n = 30	Established RA n = 30	Healthy Control n = 15	p-value*
LVEDV indexed, ml/m^2^	81±15	76±13	87±14	**0.07**

LV Mass indexed, g/m^2^	43±9	33±8	41±9	**<0.01**

LV Mass/LVEDV, g/ml	0.54±0.11	0.44±0.09	0.48± 0.12	**<0.01**

LVEF, %	60±4	59±4	61±5	0.90

Ecc Mid Ventricle	-0.22±0.03	-0.22±0.05	-0.24±0.04	0.22

Data presented as mean+/-SD unless otherwise stated. *Appropriate parametric/non-parametric tests applied for 3-way comparison after normality assessments. Ecc; Peak systolic strain, EDV; End-diastolic volume, EF; Ejection fraction.

## Conclusions

We compare two distinct RA populations and demonstrate that ventricular systolic performance (LVEF and Ecc) in the RA population is similar to controls of similar age. Abnormal LV geometry (LV mass and LVmass/EDV) is prevalent in asymptomatic patients with established RA. LV remodelling appears to occur despite excellent disease control, with the caveat that this is cross-sectional disease activity. These insights suggest that ventricular remodelling occurs later in RA and that CMR is a sensitive tool to detect such changes. Additional longitudinal evaluation can inform on the impact of inflammation/disease activity over time.

## Funding

The study is funded by an EME year project grant (11/117/27).

JPG and SP receive a research grant from Philips Healthcare.

SP is funded by British Heart Foundation fellowship (FS/10/62/28409).

